# Singing strategies are linked to perch use on foraging territories in heart‐nosed bats

**DOI:** 10.1002/ece3.8519

**Published:** 2022-02-11

**Authors:** Grace C. Smarsh, Ashley M. Long, Michael Smotherman

**Affiliations:** ^1^ Department of Brain Sciences Weizmann Institute of Science Rehovot Israel; ^2^ Department of Biology Texas A&M University College Station Texas USA; ^3^ Agricultural Center and School of Renewable Natural Resources Louisiana State University Baton Rouge Louisiana USA

**Keywords:** *Cardioderma cor*, foraging strategy, heart‐nosed bat, singing, social behavior, space use, territoriality

## Abstract

Acoustic communication allows animals to coordinate and optimize resource utilization in space. *Cardioderma cor*, the heart‐nosed bat, is one of the few species of bats known to sing during nighttime foraging. Previous research found that heart‐nosed bats react aggressively to song playback, supporting the territorial defense hypothesis of singing in this species. We further investigated the territorial defense hypothesis from an ecological standpoint, which predicts that singing should be associated with exclusive areas containing a resource, by tracking 14 individuals nightly during the dry seasons in Tanzania. We quantified the singing behavior of individuals at all perches used throughout the night. Using home range analysis tools, we quantified overall use, night ranges and singing ranges, as well as areas used in early and later time periods at night. Males sang back and forth from small ($\bar{x}$ = 3.48 ± 2.71 ha), largely exclusive areas that overlapped with overall night ranges used for gleaning prey. Individuals varied in singing effort; however, all sang significantly more as night progressed. Subsequently, areas used earlier at night and overall use areas were both larger than singing areas. Individuals varied in singing strategies. Some males sang for long periods in particular trees and had smaller core areas, while others moved frequently among singing trees. The most prolific singers used more perches overall. Our results support the hypothesis that acoustic communication repertoires evolved in support of stable foraging territory advertisement and defense in some bats.

## Graphical abstract

Heart‐nosed bats sing loud, audible songs back and forth from perches. Space use analyses demonstrate that singing occurs on discrete foraging territories, supporting the use of singing to advertise and maintain territories. Males vary in singing patterns and perch use, but more prolific singers use more perches, and have smaller core areas.

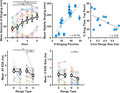

## 1 INTRODUCTION

Vocal signaling can be used by territorial individuals to defend resources such as food, mates, and roosts (Hinde, 1956; Tinbergen, 1957), and may encode important information to conspecifics regarding the signaler's identity, age, sex, location, motivational state, energetic condition, and more (Bradbury & Vehrencamp, 2011). Singing is a common signaling mechanism used by songbirds to defend resources (Catchpole & Slater, 2008). Songs range from simple to complex, and can change in duration and rate (Cardoso, 2014; Funghi et al., 2015; Linhart et al., 2013), composition (DuBois et al., 2009; Galeotti et al., 1997), or type (Akçay et al., 2013; Stoddard, 1992) to express heightened motivation during territorial contests, thus contributing to the fitness of individuals (Catchpole & Slater, 2008); carrying capacity of populations (Ahlering & Faaborg, 2006); maintenance of local populations in fragmented, degraded, or restored landscapes (Campomizzi et al., 2008); and distributions of territories or home ranges (Farrell et al., 2012). Given sampling bias and technical constraints, the degree to which non‐avian taxa use singing as a behavioral mechanism to coordinate and optimize resource utilization, particularly access to foraging opportunities, is still relatively unknown. However, research on mammals such as gibbons (e.g., Ham et al., 2016) and rodents (e.g., Pasch et al., 2013) has demonstrated that animal use of vocalizations classified as songs to maintain or defend territories extends beyond birds.

Primarily nocturnal, bats rely heavily on acoustic signals for survival, including echolocation to navigate and locate prey, and various social calls for behavioral interactions (Altringham & Fenton, 2003). Their broad communication repertoires include singing, which has been observed in five families (Smotherman et al., 2016; Smotherman et al., 2016). Although there are over 1400 species of bats (Simmons & Cirranello, 2020), we know very little about how bats use vocal communication, including singing, as a spacing mechanism or to defend resources. Territoriality is established from an ecological standpoint (home range analysis showing repeat use of an exclusive area), and a behavioral standpoint (defensive behavioral interactions) (Burt, 1943; Maher & Lott, 1995). Studies on bats generally focus on either the ecology (Conenna et al., 2019; Egert‐Berg et al., 2018; Hillen et al., 2009; Winkelmann et al., 2003) or behavior of the species (Barlow & Jones, 1997; Götze et al., 2020; Rydell, 1989; Wright et al., 2014). We examined territoriality in bats from both an ecological and a behavioral standpoint by quantifying the spatial and temporal relationships between singing behavior and foraging areas used by heart‐nosed bats (*Cardioderma cor*), one of the few bat species known to sing during nighttime foraging bouts (McWilliam, 1987; Smarsh & Smotherman, 2015a, 2017; Vaughan, 1976).

The heart‐nosed bat is endemic to East Africa (Vaughan, 1976). They use quiet echolocation to navigate, but ultimately rely on prey‐generated noises to glean frogs, beetles, and other arthropods off surfaces. Individuals forage by perching in *Acacia* trees and bushes listening for prey items nearby (Kaňuch et al., 2015; Ryan & Tuttle, 1987; Smarsh & Smotherman, 2015b), a passive gleaning strategy that is often associated with dispersed and defensible food resources (Egert‐Berg et al., 2018). Researchers have observed individuals in Acacia trees broadcasting loud, audible songs from foraging areas (McWilliam, 1987; Smarsh & Smotherman, 2015a; Vaughan, 1976, Figure S1). Singing is described by both structure of the signal and behavioral context. Similar to birds, *C. cor's* songs are multisyllabic with multiple syllable types produced with an underlying sequence pattern, and are produced in bouts at a characteristic pattern of the day. These acoustic features meet Catchpole and Slater's definition of singing in birds (Catchpole & Slater, 2008; Smarsh & Smotherman, 2015a, 2017, Figure S2). Singing is further described functionally as a seasonal behavior produced during the courtship season for breeding and territorial defense (Catchpole & Slater, 2008). *C. cor* singing has been noted to be produced during the long dry season, when prey availability is low, and appears to be a male‐specific behavior (McWilliam, 1987; Smarsh & Smotherman, 2017). Responses to song playback on *C. cor* foraging areas showed that heart‐nosed bats actively defend their nocturnal perches using their individualistic songs (Smarsh & Smotherman, 2015a, 2017). In the morning, heart‐nosed bats return to their communal day roosts, which are often located in the cavities of baobab trees (*Adansonia digitata*), and in some regions within caves and abandoned buildings (Csada, 1996). Colony size ranges generally between 5 and 100 conspecifics (Vaughan, 1976).

We hypothesized that heart‐nosed bats sing to advertise, maintain, and defend their food resources on a discrete, exclusive territory and, based on criteria for territoriality, we predicted that (1) singing areas should occur in the same locations as food resources, (2) foraging areas should be used repeatedly by the same individual, and (3) foraging areas should have minimal overlap with neighbors (Burt, 1943; Maher & Lott, 1995). We used telemetry data and behavioral observations of heart‐nosed bats from our study site in Tanzania to link foraging areas with singing locations. We examined nightly variation in singing behavior, the overlap between home ranges and singing areas, and the extent of spatial overlap between neighbors. Furthermore, we examined variation in individual singing and perch use to understand how behavior may influence space use.

## 2 MATERIALS AND METHODS

### 2.1 Study area

**FIGURE 1:**
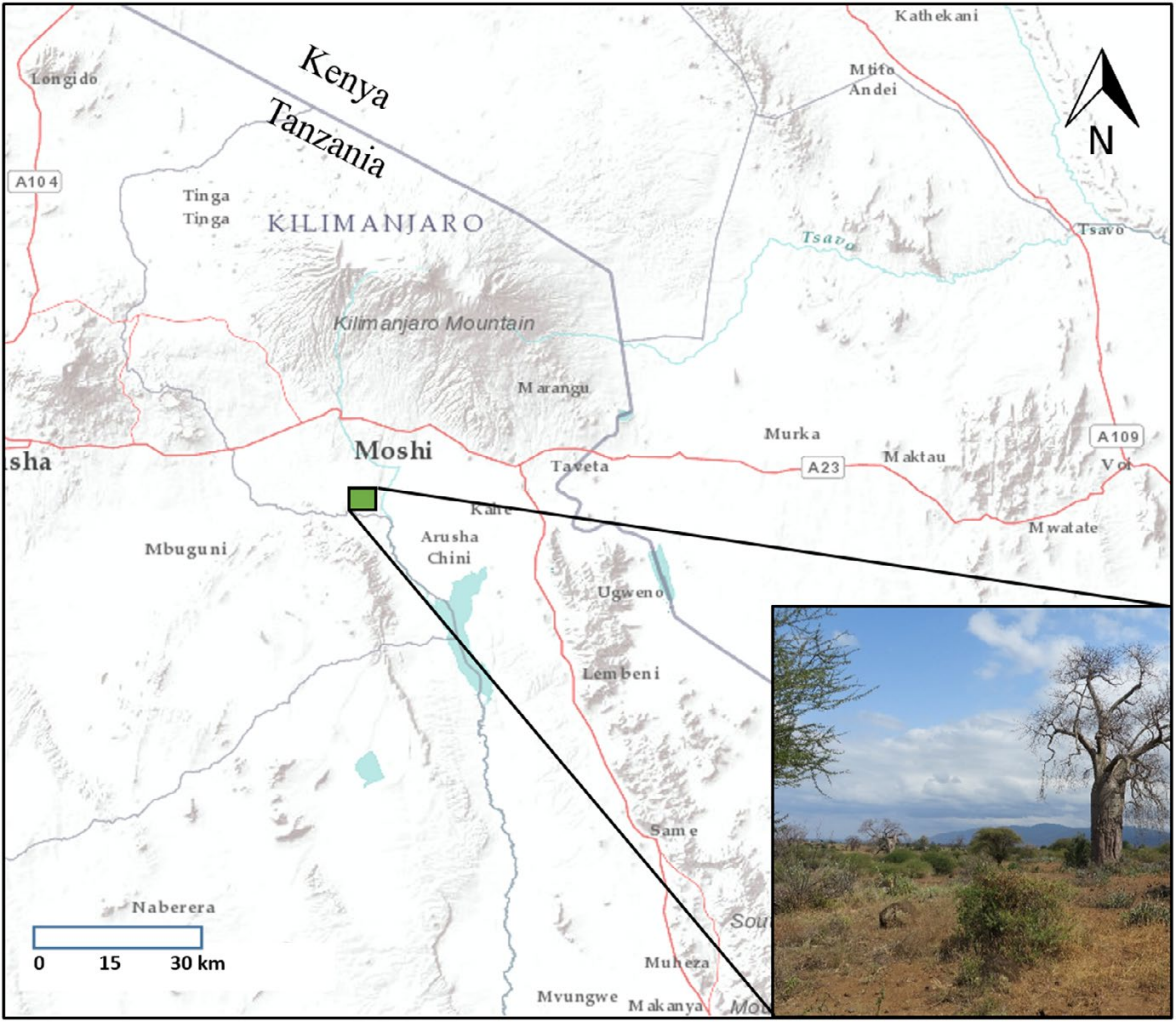
Field site location (green square) in Tanzania, characterized by acacia‐scrub habitat

We conducted our research in the open areas of the Kikavuchini, Mkalama, and Longoi Villages in the Hai District of northern Tanzania (3°27′18.324″S, 37°16′51.312″E; Figure 1). This rocky, dry habitat is characterized by Acacia‐Commiphora scrub vegetation (*A. tortilis* and *Commiphora africana*) scattered with baobab trees and is fragmented by agricultural fields. We worked in the vicinity of a known heart‐nosed bat roost of ~70–80 individuals of mixed sex and age located within a baobab tree. Mean yearly temperature in the region is 23.4°C and mean yearly precipitation is 856 mm. There are two rainy seasons each year (March–May and November–December), with the greatest amount of precipitation in April (mean 282 mm) and the least amount of precipitation in August (mean 14 mm). We conducted our research under Texas A&M University ethics AUP 2012‐087; and Tanzania Commission for Science and Technology 2014‐53‐ER‐2012‐58, 2013‐65‐NA‐2012‐58, and NA‐2012‐58.

### 2.2 Target netting and tagging individuals

In 2013 and 2014, we target‐netted heart‐nosed bats at singing perches and at one roost within our ~1500 ha study area. Because previous research found that heart‐nosed bat singing is most prevalent during the long, dry season (May–October), we focused our sampling efforts within this time period (McWilliam, 1987; Vaughan, 1976). We located individuals to net based on aural detections of their loud, conspicuous songs (McWilliam, 1987; Smarsh & Smotherman, 2015a; Vaughan, 1976). We deployed single‐high mist nets around trees that we observed were frequently used for singing (38 mm mesh, 75‐denier/2‐ply black polyester, 2.6 m high, 4 shelves, 6 m wide from Avinet, Inc., Dryden, NY). In 2013, all of the bats we captured around singing trees were male. In 2014, we captured females by placing mist nets near the baobab roost, and deployed radiotransmitters (see below) on adult females that did not show signs of pregnancy or nursing (Brunet‐Rossinni & Wilkinson, 2009).

We recorded the following for each individual: weight (g), forearm length (mm), sex, reproductive status, and age (Brunet‐Rossinni & Wilkinson, 2009, Kunz et al., 2009). We also measured testes length and width for adult males. In 2013, we marked bats using lipped forearm bands (2.9‐mm wide, alloy, Porzana Limited) (Kunz & Weise, 2009), but given minor forearm irritation, in 2014, we used passive integrated transponder (PIT) tags (HPT8 134.2 tag, Biomark). We then affixed a radiotransmitter (Model SOPB‐2012, 1.0 g, Wildlife Materials Inc.) on the dorsal region with Ostobond (2013) or the better‐performing Permatype surgical cement (2014).

We used a 3‐element folding yagi antenna and receiver (TRX‐48, Wildlife Materials) to track individuals one at a time post roost emergence during the hours of approximately 20:00–23:00 and 00:00–03:00. We used homing with multiple readings taken around the perch to target individuals (Amelon et al., 2009), assisted by their audible singing. We marked perches with a Global Positioning Systems (GPS) unit (3 m accuracy; Magellan, San Dimas, CA). We gave each marked perch an identifying number and recorded how long the individual stayed at this location, and the times of movements to perches. We recorded the times and locations of singing. Individuals sometimes flew from the perch after singing clearly indicating the end of a bout, however, if the bat was silent in the same location, we identified the end of a bout when an individual stopped singing for approximately 1 min (Smarsh & Smotherman, 2015a, 2017). We calculated the mean intersong interval for 10 recorded bats (20 songs per bat) as 9.4 s, so 1 min was a conservative approximation of singing end time (Smarsh & Smotherman, 2017, Figure S2). We recorded the songs of each bat during tracking with an SM2BAT+ recorder and SMX‐US microphone (Wildlife Acoustics), held approximately 3 m from the individual (96 kHz sample rate, 64 dB gain). We used songs from 7 of the tracked individuals in an analysis demonstrating individuality at the syllable acoustic level (Smarsh & Smotherman, 2015a).

### 2.3 Data analysis

We used ArcMap v. 10.3 (ESRI, 2014) to construct Minimum Convex Polygons (MCPs) based on all the points we recorded for each individual (i.e., night range; NR hereafter) and for points recorded when we observed the bats singing (i.e., singing range; SR hereafter). We calculated Kernel Density Estimates (KDEs) for NR and SR, as well for points recorded during the early portion of the night (~20:00–23:00; ER hereafter) and the late portion of the night (~0:00–3:00; LR hereafter). We calculated the KDEs using Geospatial Modeling Environment v. 7.4.0 (Beyer, 2015) for each individual with greater than 30 points recorded over the course of our surveys (Amelon et al., 2009). Prior to creating our KDEs, we subsampled the times that bats spent at their perches by 2‐min intervals because individuals could easily cross the approximate boundaries of their territories within this time period. Finally, we calculated the area of 50% and 95% probability isopleths of each NR, SR, ER, and LR KDE (Millspaugh et al., 2012). We calculated the centroids of the 50% KDEs and calculated the percent overlap of adjacent 50% KDEs. We compared the size of NR and SR MCPs, and NR, SR, ER, and LR KDEs using matched‐pair t‐tests and Wilcoxon signed‐rank tests. We examined spatial shifts in the areas used by comparing the locations of centroids using Hotelling's T^2^ tests. Finally, we used the intersect tool in ArcMAP to calculate 2‐dimensional overlap of KDEs between neighbors.

We used the singing start and stop times we noted to calculate amount of singing. Because we used a 1 min criterion for the end of a singing bout (unless the bat flew from the perch immediately after singing), we subtracted 50 s (1 min minus the approximate mean intersong interval) from singing bout durations. On a few occasions, an individual only sang 1 to 3 songs, in which case we averaged the song duration of 15–20 recorded songs and used the average song duration and intersong interval to calculate singing duration. We summed the amount of singing time per perch, per hour, per early, and late time periods each night, and total each night. For each individual, we calculated the average amount of singing per night, the amount of time spent singing per hour averaged across nights, and the proportion of time individuals spent singing at each perch. We used repeated‐measures ANOVA and post‐hoc matched‐pair t‐tests to test for differences in the meantime bats spent singing per hour and Welch's ANOVA to singing amounts across individuals. We compared early versus late night singing, and number of singing versus total perches used with matched pair t‐tests. We used Pearson's r and Spearman's ρ to examine correlations between mean nightly singing amount and number of perches used, maximum amount of singing per perch, range sizes, and morphometric data.

## 3 RESULTS

### 3.1 Nightly behavior

We tracked 13 males (all of which sang) and one female that did not sing, but produced contact calls (Smarsh & Smotherman, 2015a; Vaughan, 1976). We tracked individuals for 4–5 nights each except for one male which was only tracked for 3 nights due to mortality from a puff adder (*Bitis arietans*) (Table 1). For two individuals on two nights (Bats 9 and 10) due to external circumstances, the 3rd hour of tracking was shifted later by one hour to the usual break time. We had fewer detections for another bat due to tag failure (Bat 13, Table 1) but collected sufficient points for KDE calculation. On average, we recorded 46 GPS points (perches) per individual (range 27–77, Table 1). All 14 individuals returned to the same area nightly during the tracking period and repeatedly visited these same perches. The mean number of sampled points we used for KDE analysis was 493 (range 111–673, Table 1). Based on our KDE minimum point criterion (*n* = 30), we calculated MCPs and KDEs for all individuals for all range types except one (Table 1). Site fidelity extended beyond the tracking period, and we recaptured nine individuals within two months after the radio transmitters ceased functioning and fell off. The perch trees that bats visited included *A. greggii*, *A. tortilis*, *A. mellifera*, *Boscia* spp., *Sclerocarya* spp., *Terminalia* spp., *Balanites aegyptiaca*, *Ehretia* spp., *Albizia* spp., and *Euphorbia tirucalli*. One bat used the sides of buildings.

**TABLE 1:** Night ranges (NR) and singing areas (SR) of tracked bats

Area of Night Range (NR) in hectares								Area of Singing Range (SR) in hectares				
Bat	Mo‐Yr tracked	Num nights tracked	Num KDE points (*n*)	95%	50%	Num MCP Points (*N*)	MCP	Num KDE points (*n*)	95%	50%	Num MCP Points (*N*)	MCP
1	Apr−14	5	111	11.41	2.36	40	10.62	N/A	N/A	N/A	N/A	N/A
2	May−14	5	673	6.48	0.76	35	10.66	331	2.69	0.36	18	1.99
3	May−14	5	385	4.04	1.02	53	3.91	100	2.23	0.48	25	1.89
4	May−14	5	592	2.77	0.19	50	4.29	517	1.45	0.11	36	3.073
5	Jun−14	4	579	2.94	0.77	46	2.25	459	3.01	0.78	40	2.086
6	Jun−14	4	621	3.24	0.89	77	3.03	390	2.63	0.66	58	2.37
7	Jun−14	4	661	0.97	0.11	38	2.61	591	0.67	0.085	36	2.42
8	July−14	5	793	1.59	0.25	49	1.36	724	1.502	0.21	49	1.357
9	July−13	3	343	3.49	0.74	47	2.76	291	3.34	0.71	36	2.69
10	July−13	5	522	3.97	0.54	45	3.18	448	3.23	0.42	21	2.82
11	Aug−13	5	633	0.67	0.08	28	1.14	403	0.39	0.027	38	1.12
12	Sept−13	4	334	2.48	0.65	60	2.27	2	N/A	N/A	N/A	N/A
13	Sept−13	5	147	2.92	0.84	28	1.68	44	1.15	0.26	15	0.84
14	Oct−13	5	508	1.78	0.35	38	4.66	88	1.21	0.19	14	0.84
Mean		4.5	493	3.48	0.68	45.5	3.89	337.5	1.96	0.36	32.17	1.96
*SD*		0.64	201.9	2.71	0.57	13.03	3.04	223.5	1.02	0.26	13.79	0.77

Note

*Bat 1 is the female who never sang. Bat 12 sang very little, and therefore did not meet the point threshold for KDE area calculation of singing area.

**FIGURE 2:**
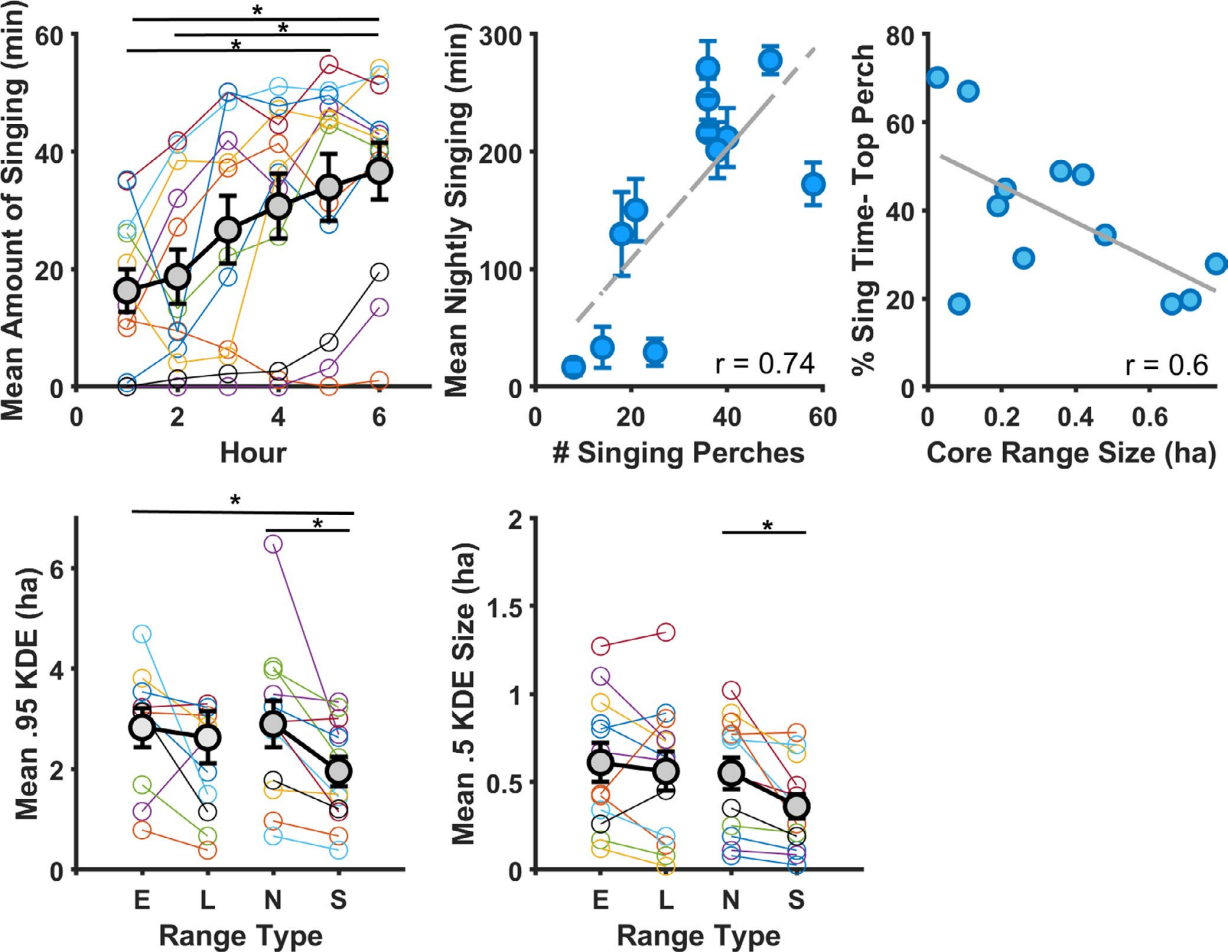
Top row: Singing increased as night progressed (black = overall means + SE; colored lines = individual means per hour). Mean ± SE amount of singing per night per individual correlated with number of singing perches. Higher percentage of time spent singing at preferred perches was negatively correlated with core range size. Bottom row: E, Early range; L, late range; N, total night range; S, singing range. 95% and 50% night ranges, and 95% early ranges, were significantly larger than singing ranges

Except for one male that largely stopped singing during our sampling period (Bat 12, not included in Tables 2 and 3), individuals foraged during early evening hours, performing short sallies from trees and audibly chewing, and occasionally singing bouts of songs from perches. The amount of singing increased hourly throughout the night (*F*_5,55_ = 10.59, *p* < .01, η^2^ = 0.17; Figure 2, Table 2). Singers sang more in the later period of the night than the earlier period of the night (*t*_11_ = −4.29, *p* < .01, *d* = 1.24). The average amount of nightly singing varied across individuals, between 16.7 min ± 13.46 and 277.73 min ± 26.48 per night (*F_11_*_,_ *_14_*_._ *_6_* = 53.9, *p* < .001, ω^2^ = 0.91, Table 3). The total number of perches used during the tracking period was greater than the number of singing perches (*t*_11_ = 2.20, *p* < .01, *d* = 1.48; Figure 2, Table 3). We tracked the most prolific singers during the middle of the dry season (June–July, Table 3). More prolific singers used more singing perches (*r* = .71, *p* < .01, Figure 2), but not more perches overall (*r* = .24, *p* = 0.43). More prolific singers had smaller testes (*r* = −.59, *p* < .05). Forearm length did not correlate with average nightly singing and perch use (*r_FA_*_‐_ *_MeanS_* = −.25, *p* = .44; *r_FA_*_‐_ *_SPerches_* = −.54, *p* = .072).

**TABLE 2:** Post hoc t‐tests comparing average amount of singing by hour of night

Contrast	Mean difference (min)	Percent increase	*T*_11_	*p* < //
Hour 1–2	2.37	114.5	0.59	.56
Hour 1–3	10.36	163.3	2.71	.02
Hour 1–4	14.35	187.7	3.48	<.01
Hour 1–5	17.6	207.5	4.85	<.01
Hour 1–6	20.35	224.3	5.35	.01
Hour 2–3	7.99	142.6	2.404	.04
Hour 2–4	11.97	163.9	2.84	.02
Hour 2–5	15.22	181.2	3.38	<.01
Hour 2–6	17.97	195.9	4.702	<.01
Hour 3–4	3.987	114.9	1.23	.24
Hour 3–5	7.24	127.1	1.96	.08
Hour 3–6	9.98	137.4	2.705	.02
Hour 4–5	3.25	110.6	1.301	.22
Hour 4–6	5.99	119.5	3.06	.01
Hour 5–6	2.75	108.1	1.35	.21

**TABLE 3:** Singing behavioral data for 12 tracked males

Bat	Mo‐year tracked	Singing‐ early period ($\bar{x}$ ± SD min)	Singing‐ late period ($\bar{x}$ ± SD min)	# Singing perches	% Singing perches	% Singing time per perch ($\bar{x}$ ± SD)*	% Singing time‐ top perch	Top perch type	Height class (m)
2	May−14	25.89 ± 32.69	104.12 ± 56.98	18	51.4	5.55 ± 11.88	48.92	*A. tortilis*	5–10
3	May−14	27.17 ± 23.76	139.76 ± 192.24	25	47.2	4.0 ± 7.11	34.44	*A. tortilis*	5–10
4	May−14	97.67 ± 43.86	146.72 ± 14.16	36	72.0	2.77 ± 11.08	67.07	*A. tortilis*	5–10
5	June−14	87.81 ± 50.95	124.1 ± 39.49	40	86.9	2.56 ± 5.32	27.86	*A. tortilis*	5–10
6	June−14	61.66 ± 42.35	110.67 ± 16.66	58	75.3	1.72 ± 2.96	18.77	*A. tortilis*	3–5
7	June−14	116.51 ± 31.7	154.53 ±19.66	36	94.7	2.7 ± 4.69	18.78	*A. tortilis*	3–5
8	July−14	126.96 ± 25.69	150.77 ± 22.87	49	100.0	2.04 ± 6.46	44.88	*Acacia* spp.	5–10
9	July−13	94.7 ± 10.88	140.972 ± 24.18	36	76.6	2.63 ± 4.83	19.703	*A. tortilis*	5–10
10	Aug−13	88.52 ± 25.42	112.81 ± 31.51	38	90.9	2.5 ±7.88	48.11	*A. tortilis*	5–10
11	Aug−13	25.46 ± 26.17	124.9 ± 49.9	21	75.0	4.76 ± 15.16	70.16	*A. tortilis*	3–5
13	Sept−13	2.803 ± 5.607	16.66 ± 13.39	8	53.6	12.5 ± 11.43	29.18	*A. mellifera*	5–10
14	Oct−13	3.59 ± 2.74	29.7 ± 37.22	14	36.8	7.14 ± 11.96	41.05	*A. mellifera*	3–5
*Mean*		62.9	101.52	31.6	71.7	4.24	39.1		
*SD*		44.71	54.2	12.2	20.3	3.06	17.6		

**FIGURE 3:**
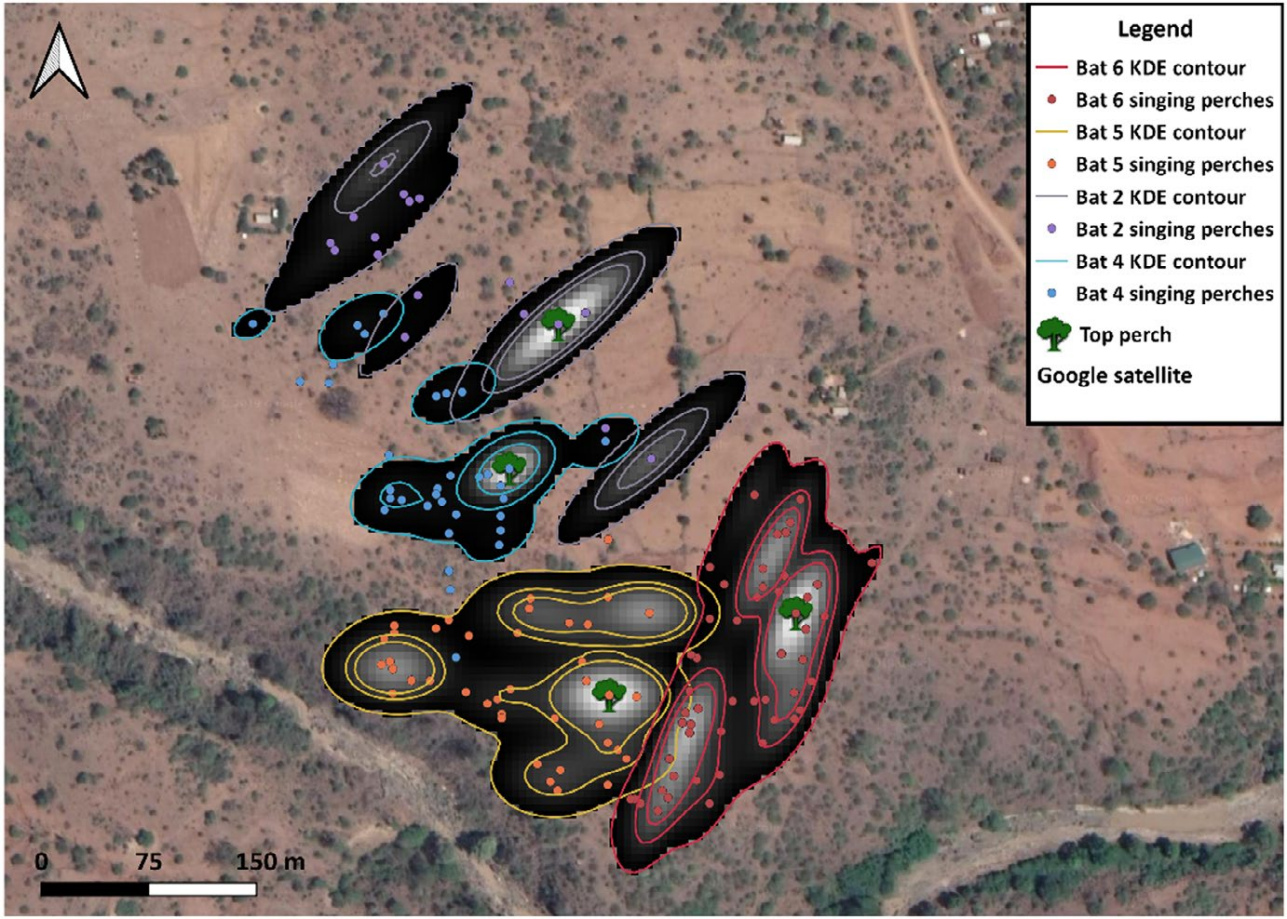
Singing range (SR) kernel density 50%, 70%, and 95% probability contours with heat map rasters of area use for four neighboring males. Lighter areas represent greater use. Top Perch symbols represent the tree where the individual spent the maximum percentage of singing time, which varied across individuals (e.g., Bats 4 and 2, top perch use = 67% and 48.9% total singing time, vs. Bats 5 and 6; top perch use = 18.7% and 27.86% total singing time). Bats who sang more in favored trees had more concentrated heat use maps and smaller core areas

Individuals varied in their singing behavior, either spending the majority of their singing time at particular perches (e.g., Bat 11 spent 70% of his singing time at one perch; Table 3, Figures 2 and 3) or using perches more evenly for singing (e.g., Bat 6 spent 19% of his singing time maximum at one perch; Table 3, Figure 3). We found no relationship between average nightly singing and the maximum percent of time spent singing at a single perch (*ρ* = −.09, *p* = .78).

### 3.2 Night range sizes based upon use and time of night

**FIGURE 4:**
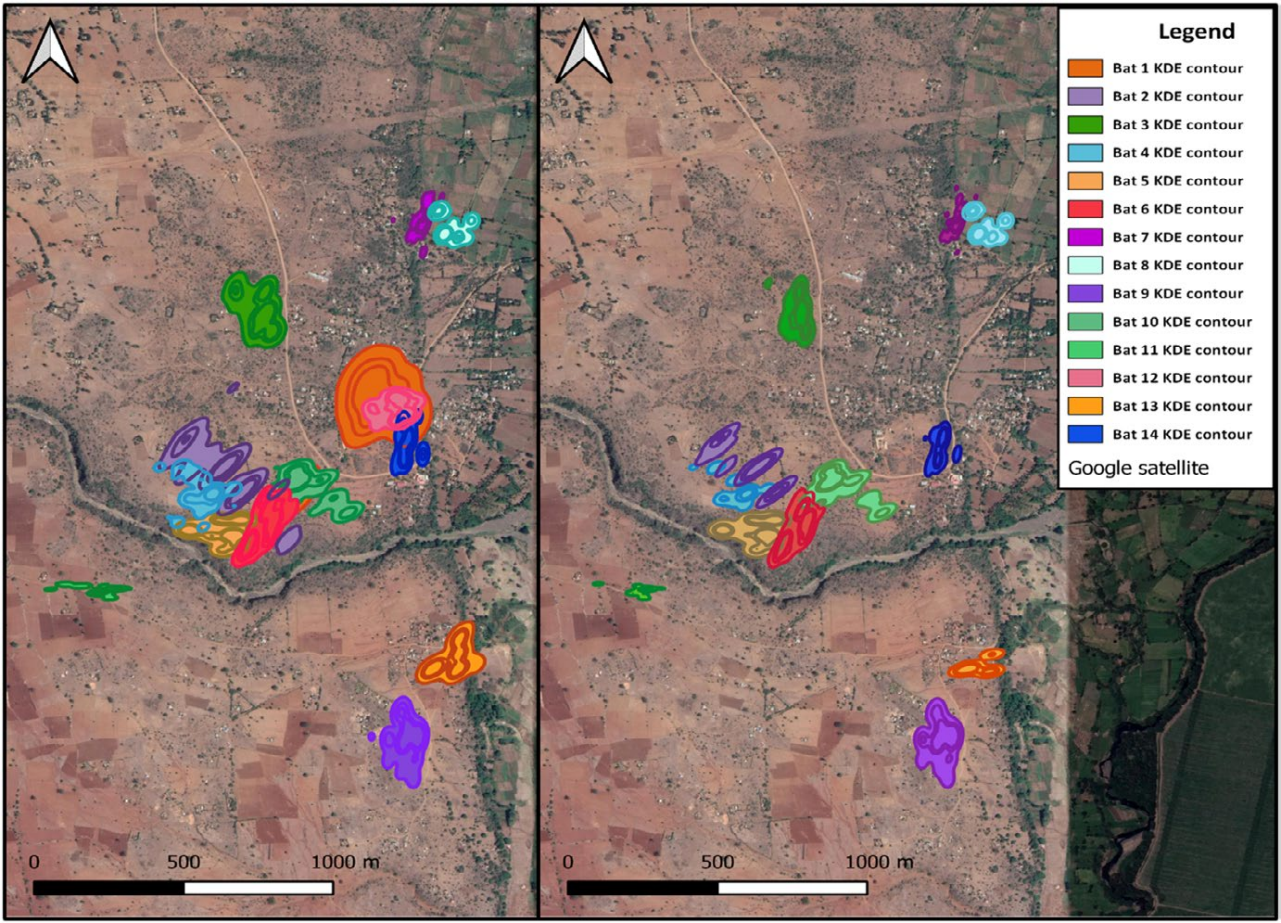
Kernel density analysis 50%, 70%, and 95% probability use contours for all bats tracked in 2013 and 2014. Left Panel—Night ranges (NR) calculated from all observations. Right Panel—Singing ranges (SR) of males calculated from points when the bat was singing. Ranges had little neighbor overlap—Greatest NR overlap was female Bat 1 with neighboring males

The night ranges (*n* = 14) calculated from minimum convex polygons (MCP‐NR) varied between 1.14 ha and 10.62 ha (Table 1), and were ~1.75 times larger than the singing ranges (MCP‐SR) (*z* = 2.31, *r* = .47 Table 1). Average nightly singing did not correlate with MCP‐NRs (ρ_AveS‐MCPNR_ = −.26, *p* = .42) or MCP‐SRs (ρ_AveS‐MCPSR_ = .50, *p* = .1; Table 1). The areas we calculated from the 95% isopleths for all points (NR) varied from 0.97 ha to 11.4 ha (Table 3, Figures 2 and 4). The mean 95% NRs were ~1.75 times larger than SRs (*t_11_* = 2.201, *p* < .01, *d* = 0.86; Table 1, Figures 2 and 4). Core NRs were 1.9 times larger than core SRs (*t_11_* = 3.201, *p* = .01, *d* = 0.89; Table 1, Figures 2 and 4). However, centroid coordinates did not shift in the location of NRs and SRs (${\bar{x}}_{\text{AbsDiffLongitude}}$ = 8 ± 11 m, ${\bar{x}}_{\text{AbsDiffLatitude}}$ = 10 ± 13 m, *T^2^* = 0.62, *F_(2_*_,_ *_10)_* = 0.28, *p* = .76, Figures 2 and 4). The amount of nightly singing did not correlate with SR or NR (*ρ*_MeanS‐.95SR_ = .14, *p* = .66; *ρ*_MeanS‐.5SR_ = 0.032, *p* = .92; *ρ*_MeanS‐.95NR_ = −.36, *p* = .26; ρ_MeanS‐.5NR_ = −.55, *p* = .07). However, bats that spent more time singing in particular perches had smaller core singing areas (*r* = −.6, *p* = .04, Figure 2).

We found no difference in the size of the ranges used early in the night (ER) or later at night (LR) (*Z* = 0.19, *p* = .86, *r* = .036; Median._5ER_ = 0.61ha, Median._5LR_ = 0.63 ha, *Z* = 0.69, *p* = .5, *r* = .691;, Figure 2), nor were these areas shifted spatially according to centroid comparison (${\bar{x}}_{\text{AbsDiffLongitude}}$ = 18 ± 15 m, ${\bar{x}}_{\text{AbsDiffLatitude}}$ = 31 ± 32 m, *T^2^* = 1.908, *F_2_*_,_ *_12_* = 0.88, *p* = .44). LR and SR differed in size (*t_11_* = 2.201, *p* = .08, *d* = 0.56; *t_11_* = −1.57, *p* = .15, *d* = .45, Figure 2). 95% isopleths of ER were larger than those of SR (*t_11_* = −2.53, *p* = .028, *d* = 0.73), but not their core areas (*t_11_* = −2.124, *p* = .06, *d* = 0.61, Figure 2).

### 3.3 Neighbor proximity and overlap

**FIGURE 5:**
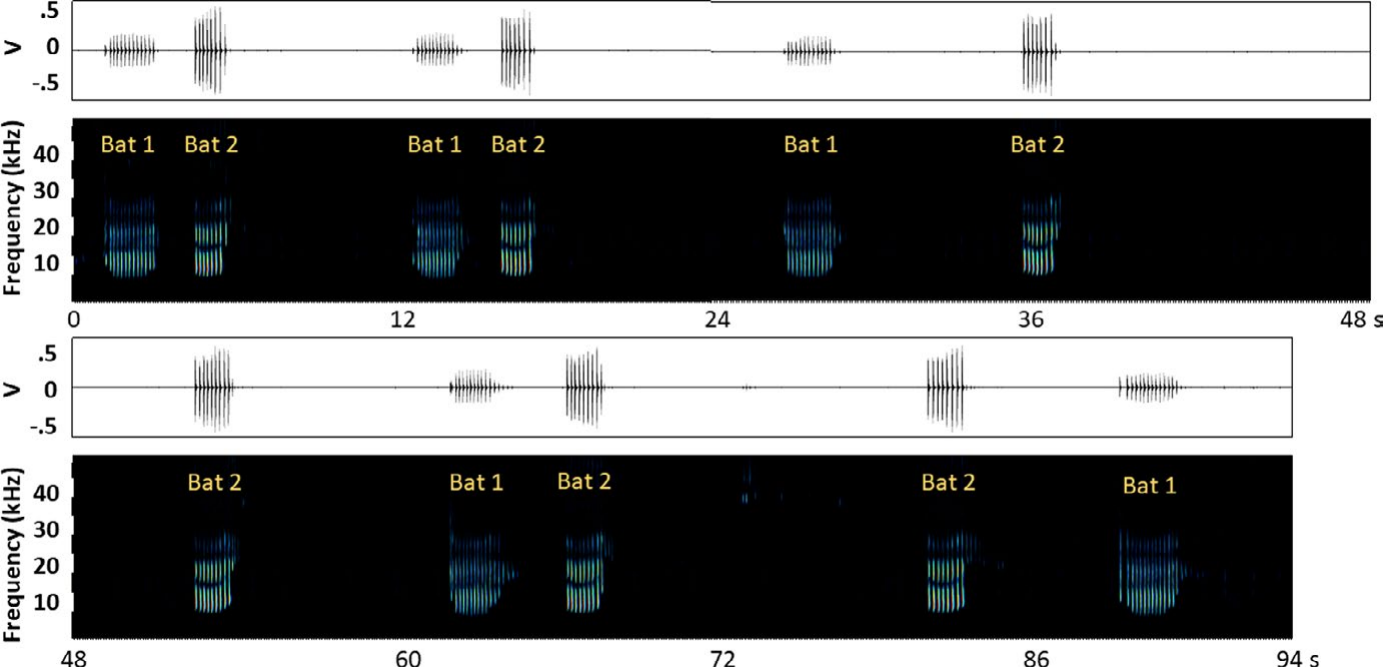
Countersinging between two males: An intruder, Bat 1, perching just within Bat 2’s territory. Note: 1 and 2 are used to differentiate songs in the figure and do not correspond to tracked bat IDs

The number of singers at the site increased as the dry season progressed, with peak numbers in June/July (*n* = 35). The number of nearest neighbors at the time of tracking varied between one and six ($\bar{x}$ = 2.4 ± 1.6). Neighbors were located adjacent to tens of meters away across treeless farming fields. On three occasions, an individual perched within 10 m of our tracked singer in the territory, resulting in counter singing until the intruder left (Figure 5). Area overlap of neighbors tracked the same year (and three individuals with known site fidelity across years) was low: There were no core SR overlaps and one core NR overlap (${\bar{x}}_{.5\text{NRoverlap}}$ = 0.1 ± 0.05%, *n* = 2, Bats 12 and 14, Figure 4). Overlap was small for 0.95 SR, ranging from 0% to 8.6% (${\bar{x}}_{.95\text{SRoverlap}}$ = 1.5 ± 2.5%, *n* = 17, Figure 4, Table S1). Neighbor pairs showed some overlap in the 0.95 isopleths of NR, ranging from 0% to 25.6% (${\bar{x}}_{.95\text{All}}$ = 5.1 ± 7.8%, *n* = 24 possible overlaps, Figure 4, Table S1), with the largest overlap between the female and a neighboring male (Figure 4, Table S1), whose NR she frequented. Only one male's NR overlapped with the NR beyond a nearest neighbor (Bats 1–2, Table S1).

## 4 DISCUSSION

### 4.1 Territoriality and social organization

As predicted under the territory defense hypothesis (Burt, 1943; Maher & Lott, 1995) and confirming previous observations (McWilliam, 1987; Smarsh & Smotherman, 2017; Vaughan, 1976), tracking *Cardioderma cor* revealed that males sing on small territories containing food sources, they return to these locations nightly, and there is minimal overlap between neighbors. This space use strategy is consistent with predictions for terrestrial gleaning species (Egert‐Berg et al., 2018). Some of the ranges we calculated from our telemetry data were larger than those estimated from observation only (Vaughan, 1976). While *C. cor* territory locations and boundaries can be reliably determined by observations of singing perches, this method may underestimate total space use, as demonstrated in Swainson's warbler (*Limnothlypis swainsonii*) (Anich et al., 2009). Scaling laws and diet can influence foraging range size (Haskell et al., 2002). *C. cor* night ranges were similar in size to the congeneric gleaning species *Megaderma lyra*, the greater false vampire bat, which is a bat of similar size and diet to *C. cor* (Audet et al., 1991), and *Lavia frons*, the yellow‐winged bat, of similar size with a diet of primarily aerial insects (Conenna et al., 2019; Vaughan & Vaughan, 1986).

The core areas of the night ranges are the focal spots for singing in *C. cor* males. As nights progressed and foraging activity decreased, bats spent more time on more concentrated areas as they increased singing output. The overlay of the singing ranges and overall use ranges (including foraging) further supports singing as a territorial behavior for resource defense foraging strategies in *C. cor*, rather than an exploded lek (Toth & Parsons, 2013). Previous work found that song playbacks conducted within the outermost singing perches of heart‐nosed bats evoke strong territorial response, but not beyond these perches, demonstrating boundary maintenance demarked by singing (Smarsh & Smotherman, 2017). Our observations of counter singing when a neighbor sang within the outer singing perches also support this mechanism of spatial organization. During the summer rains, singing ceases and males disperse (Vaughan, 1976), but opportunistic recapture data from this paper and others suggests that territory fidelity of heart‐nosed bats extends across years (McWilliam, 1987; Vaughan, 1976).

Similar to the multi‐use territories in songbirds and gibbons (Ham et al., 2016; Marshall & Marshall, 1976; Mitani, 1984, 1985, 1987; Raemaekers & Raemaekers, 1985), McWilliam noted that male–female heart‐nosed bat pairs hold territories, which was not observed in this study (McWilliam, 1987). A sympatric species, *Lavia frons*, the yellow‐winged bat, has multi‐use territories in which male–female pairs roost in Acacia trees and forage on the territories (Conenna et al., 2019; Vaughan & Vaughan, 1986; Wickler & Uhrig, 1969, Pers. Obs.). For *C. cor*, our study suggests that females have fidelity to foraging areas that may overlap more with neighboring males, and do not sing. On several occasions, we observed a non‐singing adult producing contact calls and joining the tracked male for short time periods, possibly for courtship, although alternatively mating would take place in the mixed‐sex colonies in baobabs. A targeted tracking study of females along with courtship observation will determine whether *C. cor* females may benefit from mating outside of the roost, such as additional access to resources, and where *C. cor* may fall on the resource defense polygyny‐ exploded lek continuum (Alonso et al., 2012; Kotrschal & Taborsky, 2010; Toth & Parsons, 2013).

### 4.2 Male singing strategies

We observed patterns of singing by night and season. Additionally, we observed variable singing effort across individuals, and more interestingly, varying strategies of singing in relation to space use. Multiple ecological and social factors can influence singing effort. The variation in singing effort across the six month dry season supports seasonality of this behavior, aligning with previous observations (McWilliam, 1987; Vaughan, 1976). For songbirds and singing mammals (Brenowitz, 2004; Coudrat et al., 2015; Smith et al., 1997; Smotherman, Bohn, et al., 2016; Smotherman, Knörnschild, et al., 2016), singing effort is seasonal and regulated by environmental cues such as temperature and daylight, and subsequent physiological changes such as testosterone levels (Nelson et al., 1990). Additional variation in singing output can relate to male fitness. Male sac‐winged bats (*Saccopteryx bilineata*) with lower frequency buzzes in their territory songs have higher fitness (Behr et al., 2006). For the lekking lesser short‐tailed bat, *Mystacina tuberculata*, smaller males have greater song output and higher fitness (Toth & Parsons, 2018). We observed that *C. cor* males with smaller testes sang more, potentially as a tradeoff for energetic output.

Beyond singing effort, we observed two main singing and space use strategies: individuals spending a large proportion of singing at particular trees or spending small amounts of time singing at more trees. The latter strategy is a reflection of more movement around the territory and resulted in larger core areas of use. These strategies could be influenced by social factors including the location and proximity of neighbors, and ecological factors including the amount of cover, and the type and height of trees on the territory. Exposed perches increased the energetic cost of singing due to higher thermoregulatory costs in willow warblers (Ward & Slater, 2005). Tree type and habitat can influence the transmission ability of songs through the habitat (Blumenrath & Dabelsteen, 2004), and has been shown to affect the decisions of animals while choosing perches. Chaffinches, for example, prefer to sing in the upper canopy of pines for better transmission of songs (Krams, 2001). Male black‐crested gibbons (*Nomascus concolor*) choose trees near key food and sleeping sites, but also select the highest trees on ridges or slopes for singing to increase vocal transmission (Fan et al., 2009). Kloss gibbons (*Hylobates klossii*) also choose emergent trees of the rain forest on their home ranges (Whitten, 1982). Perch height can also have an effect on social dynamics of rival territory holders. Nightingales change their singing output in response to the perceived perch height of neighbors (Sprau et al., 2012). Lastly, predation risk is a cost for loud, conspicuous signals that may influence behavior (Möller et al., 2005), such as greater perch switching (Marler, 1956). Krams (2001) found that chaffinch males move to lower canopy perches in response to sparrowhawk models (Krams, 2001). The lower frequencies of *C. cor* song syllables (between 8 and 10 Khz) (Smarsh & Smotherman, 2015a) are within the audiogram of barn owls, a bat predator that may influence behavior (Baxter et al., 2006; Lima & O’Keefe, 2013). Personality can create variability in response to predation risk, in which bolder individuals are less influenced by a predator. More explorative and risk‐taking male collared flycatchers (*Ficedula albicollis*) sing at lower perches in the presence of a human observer (Garamszegum et al., 2008). These personality traits can be consistent in individuals, regardless of body condition (Dammhahn & Almeling, 2012). The shy‐bold continuum of behavioral variability could thus be an important factor in singing and movement strategies (Wilson et al., 1994).

## 5 CONCLUSIONS

Our data provide a clear, quantitative link between the nighttime spatial patterns and communication behaviors of male *Cardioderma cor*. For a “whispering” bat using quiet echolocation, singing is likely an efficient mechanism for advertising and defending a small foraging territory rather than continually flying about or eavesdropping on the echolocation of passerby. Heart‐nosed bat singing is tightly linked to perches on foraging areas, with variation in strategy of tree use and subsequent core area size. Singing location is an excellent proxy for territory presence, but the variation in behavior and space use during the course of the dry season and throughout the night demonstrates the importance of different levels of temporal scales in habitat use studies. *C. cor* remains an intriguing species for exploring questions connecting behavior and ecology from evolutionary or conservation perspectives.

## CONFLICT OF INTEREST

We declare no conflicts of interest.

## AUTHOR CONTRIBUTIONS

**Grace C. Smarsh:** Conceptualization (lead); Data curation (lead); Formal analysis (lead); Funding acquisition (lead); Investigation (lead); Methodology (lead); Project administration (lead); Supervision (lead); Writing – original draft (lead); Writing – review & editing (lead). **Ashley M. Long:** Formal analysis (supporting); Methodology (supporting); Writing – review & editing (equal). **Michael Smotherman:** Conceptualization (equal); Funding acquisition (supporting); Investigation (supporting); Methodology (supporting); Resources (supporting); Supervision (supporting); Validation (supporting); Visualization (supporting); Writing – review & editing (equal).

## Supporting information

Fig S1

Fig S2

Table S1

## Data Availability

Data is available on Zenodo (https://doi.org/10.5281/zenodo.5711115) and can be tracked by Ref # 5711115 on zedono.org.
