# Supplementary figures and images for: Singing strategies are linked to perch use on foraging territories in heart‐nosed bats
Source: Ecol Evol. 2022 Feb 11;12(2):e8519. doi: 10.1002/ece3.8519 (PMC8837579; doi:10.1002/ece3.8519)

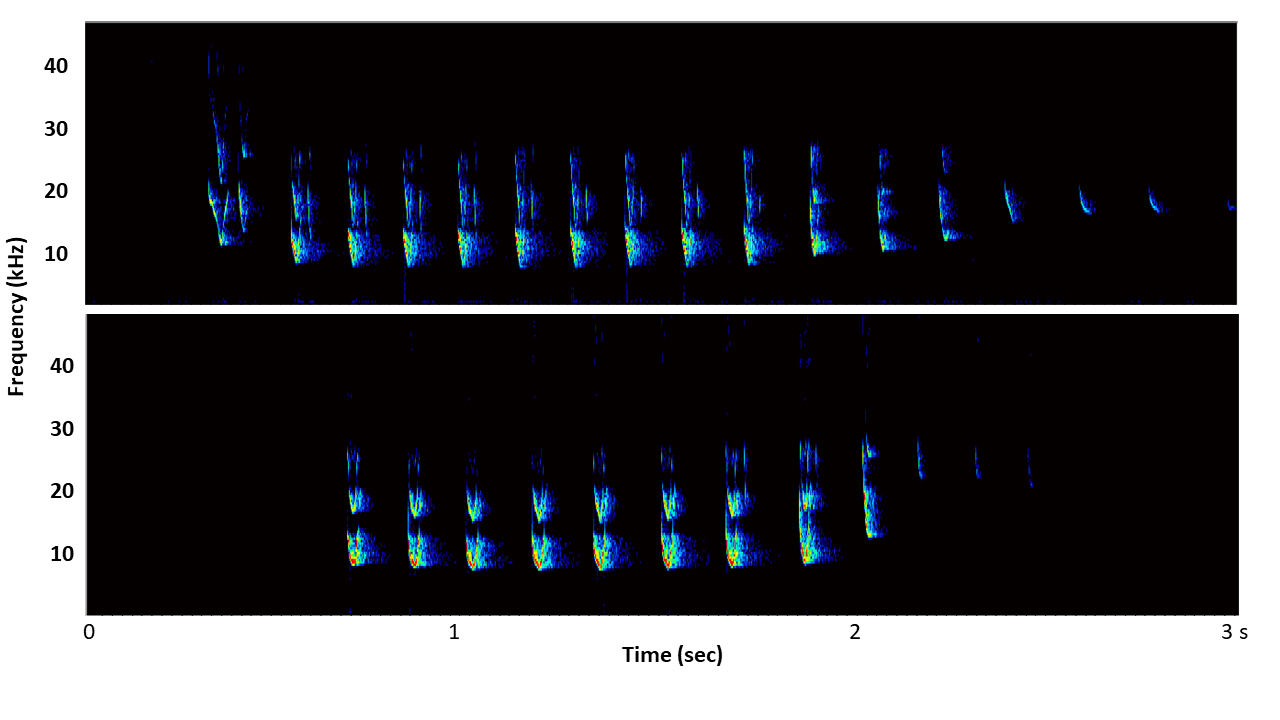

Supplement: Supplementary file 1 — Fig S1 [file ECE3-12-e8519-s004.tif]

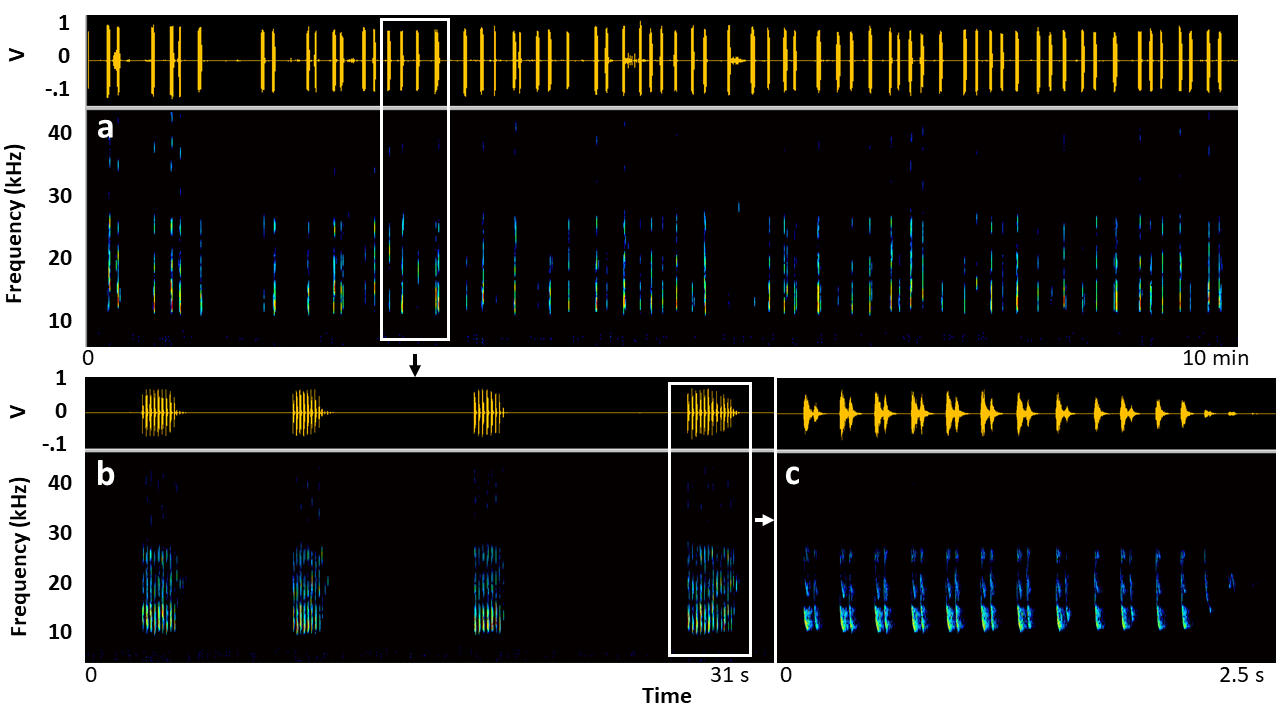

Supplement: Supplementary file 2 — Fig S2 [file ECE3-12-e8519-s001.tif]
