# Supplementary material for: Singing strategies are linked to perch use on foraging territories in heart‐nosed bats
Source: Ecol Evol. 2022 Feb 11;12(2):e8519. doi: 10.1002/ece3.8519 (PMC8837579; doi:10.1002/ece3.8519)
Supplement: Supplementary file 3 — Table S1 [file ECE3-12-e8519-s002.docx]

**Supp. Table 1**. Percent overlap between tracked bats and nearest tracked neighbors of their 95% KDE Night Ranges and Singing Ranges

| **Bat** | **Neighbor** | **NR 0.95 Overlap (%)** | **SR 0.95 Overlap (%)** |
| --- | --- | --- | --- |
| **2** | 4 | 10.9 | 4.6 |
|  | 5 | 0 | 0 |
|  | 6 | 1.3 | 0 |
|  | 1 | 0.8 | 0 |
| **4** | 2 | 25.6 | 8.6 |
|  | 5 | 3.8 | 0 |
|  | 6 | 0 | 0 |
| **5** | 2 | 0 | 0 |
|  | 4 | 3.6 | 0 |
|  | 6 | 4.3 | 4.1 |
| **6** | 2 | 2.5 | 0 |
|  | 4 | 0 | 0 |
|  | 5 | 3.9 | 4.7 |
| **7** | 8 | 1.8 | 1.3 |
| **8** | 7 | 1.1 | 0.58 |
| **9** | 13 | 0 | 0 |
| **13** | 9 | 0 | 0 |
| **12** | 13 | 3.7 | NA |
|  | 1 | 17.8 | NA |
| **14** | 12 | 5.1 | NA |
|  | 1 | 28.5 | NA |
| **1** | 13 | 4.4 | NA |
|  | 12 | 3.9 | NA |
|  | 2 | 0.43 | NA |
| **Mean** |  | 5.1 | 1.5 |
| **SD** |  | 7.9 | 2.5 |
