# Supplementary material for: Singing strategies are linked to perch use on foraging territories in heart‐nosed bats
Source: Ecol Evol. 2022 Feb 11;12(2):e8519. doi: 10.1002/ece3.8519 (PMC8837579; doi:10.1002/ece3.8519)
Supplement: Supplementary file 4 [file ECE3-12-e8519-s003.docx]

**Supp. Fig S1**: Representative songs from two different heart-nosed bats. Songs are multisyllabic and vary across individuals in syllable spectrotemporal parameters.

**Supp. Fig S2:** a) Example of a bout from a heart-nosed bat. Songs are clearly patterned, multisyllabic clusters. An individual can sing for an hour straight. b) Zoomed in section of a bout. As individuals sing the song duration varies, as well as the number and placement of syllable types. c) Example of a song.
